# Genetic analysis of geometric morphometric 3D visuals of French jumping horses

**DOI:** 10.1186/s12711-023-00837-8

**Published:** 2023-09-18

**Authors:** Anne Ricard, Nathalie Crevier-Denoix, Philippe Pourcelot, Harmony Crichan, Margot Sabbagh, Bernard Dumont-Saint-Priest, Sophie Danvy

**Affiliations:** 1 Pôle développement, innovation et recherche, Institut français du cheval et de l’équitation, 61310 Exmes-Gouffern en Auge, France; 2 Université Paris Saclay, INRAE, AgroParisTech, GABI, 78350 Jouy-en-Josas, France; 3 INRAE, Ecole Nationale Vétérinaire d’Alfort, Unité 957 BPLC, 94700 Maisons-Alfort, France

## Abstract

**Background:**

For centuries, morphology has been the most commonly selected trait in horses. A 3D video recording enabled us to obtain the coordinates of 43 anatomical landmarks of 2089 jumping horses. Generalized Procrustes analysis provided centered and scaled coordinates that were independent of volume, i.e., centroid size. Genetic analysis of these coordinates (mixed model; 17,994 horses in the pedigree) allowed us to estimate a variance–covariance matrix. New phenotypes were then defined: the “summarized shapes”. They were obtained by linear combinations of Procrustes coordinates with, as coefficients, the eigenvectors of the genetic variance–covariance matrix. These new phenotypes were used in genome-wide association analyses (GWAS) and multitrait genetic analysis that included judges’ scores and competition results of the horses.

**Results:**

We defined ten shapes that represented 86% of the variance, with heritabilities ranging from 0.14 to 0.42. Only one of the shapes was found to be genetically correlated with competition success (r_g_ = − 0.12, standard error = 0.07). Positive and negative genetic correlations between judges’ scores and shapes were found. This means that the breeding objective defined by judges involves improvement of anatomical parts of the body that are negatively correlated with each other. Known single nucleotide polymorphisms (SNPs) on chromosomes 1 and 3 for height at withers were significant for centroid size but not for any of the shapes. As these SNPs were not associated with the shape that distinguished rectangular horses from square horses (with height at withers greater than body length), we hypothesize that these SNPs play a role in the overall development of horses, i.e. in height, width, and length but not in height at withers when standardized to unit centroid size. Several other SNPs were found significant for other shapes.

**Conclusions:**

The main application of 3D morphometric analysis is the ability to define the estimated breeding value (EBV) of a sire based on the shape of its potential progeny, which is easier for breeders to visualize in a single synthetic image than a full description based on linear profiling. However, the acceptance of these new phenotypes by breeders and the complex nature of summarized shapes may be challenging. Due to the low genetic correlations of the summarized shapes with jumping performance, the methodology did not allow indirect performance selection criteria to be defined.

**Supplementary Information:**

The online version contains supplementary material available at 10.1186/s12711-023-00837-8.

## Background

Horse breeding has been based on the selection for conformation traits since the nineteenth century. Conformation traits were part of the definition of the breed standard when studbooks were created. The traditional assessment of conformation was based on subjective evaluation in relation to the breeding objective [1]. Currently, linear profiling [2] is often preferred because it describes rather than evaluates the morphology along a linear scale from one biological extreme to the other. Linear profiling has been studied in a wide variety of horse breeds, from draught horses [3–7] to sport and leisure horses [8–19], including breeds that are specialized in show jumping [20–28]. Linear profiling is considered to be the reference [29] for selection on morphology and the study of correlations with performance traits. Biometric measurements have rarely been used, except for height at withers, chest girth, and cannon bone circumference, mainly because of the difficulty of their recording, as they require handling the horses, or the use of standardized photography for more complex biometrics such as angles [30–32].

Morphology is the study of the form and structure of organisms and their specific structural features. In 2002, Pourcelot et al. [33] developed a rapid 3D video method to record the coordinates of anatomical landmarks in 3D space. Nowadays, morphology can by analyzed in a more global way than in terms of lengths and angles through “geometric morphometrics” that define morphotypes or shapes [34]. Genetic studies of these shapes have been proposed [35] and performed in Franches-Montagnes horses [36]. The objective of our study was to use 3D anatomical landmarks and geometric morphometrics for genetic and genomic analysis of the morphology of French jumping horses, defined as morphotypes called shapes. Further genetic analyses addressed the genetic correlations of shapes with conformation assessment by judges and competition performance.

## Methods

### Horses

Morphological data were recorded on 2053 young jumping horses, at the end of a show jumping competition, and on 36 of their sires. A first sample of 784 horses was recorded in 2002 and subsequent samples in 2015 (734) and in 2016 (571). Recording was performed at 24 different events, with between 15 and 201 horses per event (average: 87.0). Gender was evenly distributed: 49% females and 51% males (including 26% geldings). Age was also evenly distributed among the young horses: 52% were 4 years old and 48% were 5 or 6 years old (with only four horses aged 6). The 36 sires were between 9 and 25 years old. The breed of the horses was mostly Selle Français (SF, 91%) with a few foreign sport horses (5%), Anglo Arabs (2%), and crossed sport horses (2%). Among those that were not SF, 39% had at least one SF parent.

### Morphometric data

**Fig. 1:**
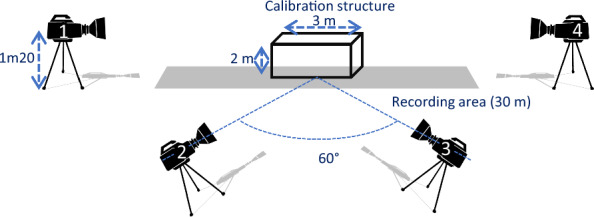
Scheme of the video protocol

**Fig. 2:**
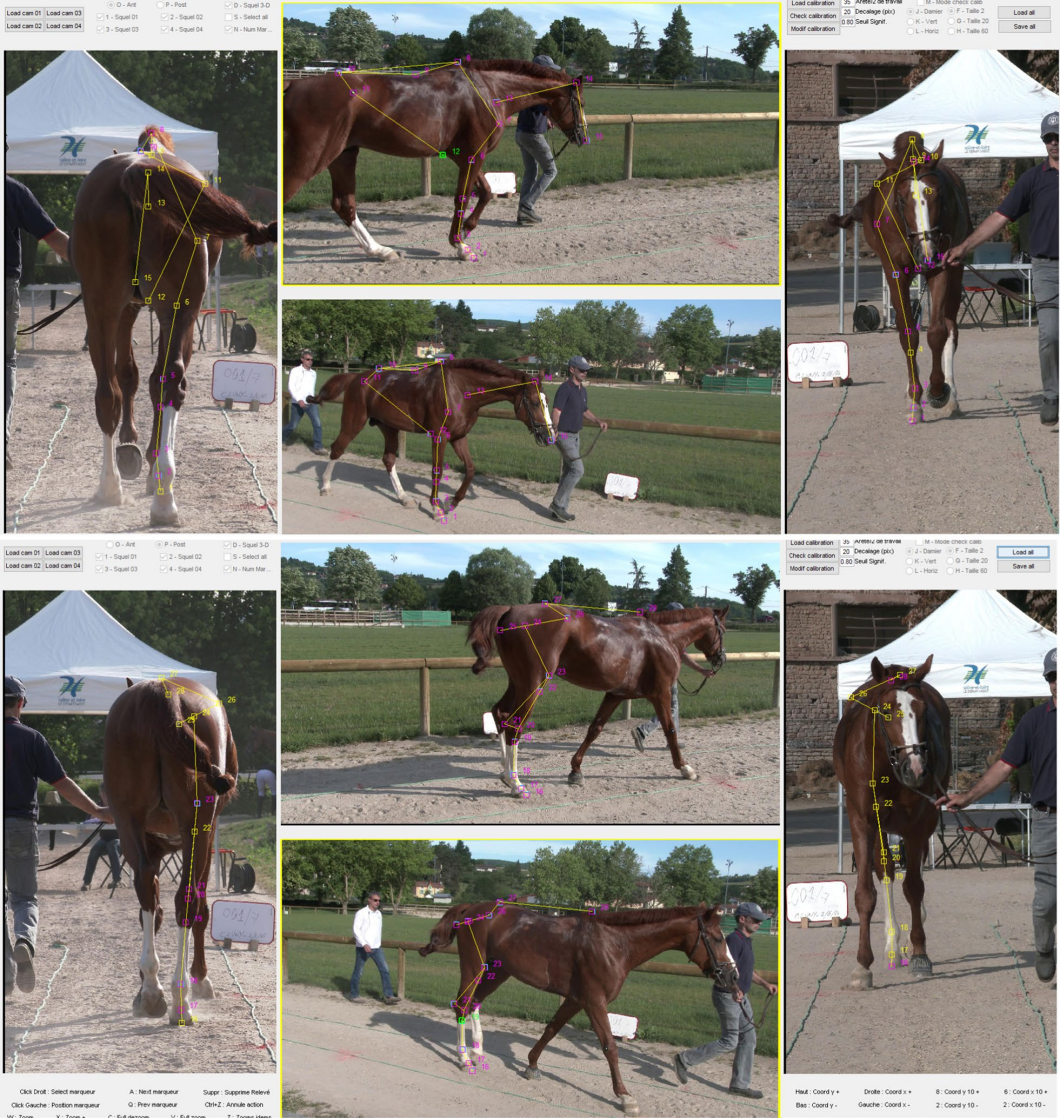
Computer images with the skeleton adjusted by the operator (forehand on top, hindquarters at bottom)

The 3D morphometric method was developed by [33, 37, 38] and has already been described in detail in [39]. Briefly, horses were filmed when led by hand while walking back and forth on a 1-m-wide and 25-m-long level track. Horses were filmed from four angles, after calibration of the cameras with an aluminum structure of known dimensions: from the front, from the back, and from two angles from the right side, slightly from the front and slightly from behind (Fig. 1). Data processing was then performed off-line with synchronized and digitized films. For each horse, two reference moments were identified: one for analysis of the forehand and one for the hindquarters. References were chosen according to the vertical position of the fore and hind cannon bone, respectively. For each reference moment, the four angle views of the horse were then downloaded onto computers. The operator then adjusted a skeleton with landmarks of the major anatomical references needed (Fig. 2), which were automatically adjusted in the three dimensions for the four views. At the end of the process, the three coordinates for each landmark were stored, resulting in 15 points for the forehand and 13 for the hindquarter, with the X, Y, and Z axes referring to the horizontal plane, the horizontal line on the transverse plane, and the vertical line on the sagittal plane, respectively.

### Competition data

Analysis of the jumping competition data was performed with the complete dataset of official jumping competitions from 2001 to 2019 since the birth year 1997, including 150,817 horses and 654,340 annual performances. Horses born abroad with a partial career in France were excluded from this dataset. Success in competition was measured by the repeated observations of an annual performance, which was the logarithm of an annual sum of points. Points were distributed in each competition according to rank and technical difficulty. Among the young horses that were measured for morphometry, nearly all (95%) performed in jumping competitions.

### Judges’ score data

Morphology was judged during young horse breeding shows organized by the French stud book of Selle Français. From 2005 to 2019, 28,412 horses aged 2 or 3 were recorded, from one to nine times per horse, with a mean of 1.6, for a total of 46,349 records. Recorded horses were 46% female. Horses were scored on a scale from 1 to 20 for nine criteria: neck, body forehand (shoulder, arm, forearm), withers-back-loins, body hindquarters (croup, pelvis, thigh), joints, stance of forelegs, stance of hindlegs, impression, and chic. Only 19% of the horses that were measured for morphometry were also judged in breeding shows but 92% of them had paternal half-sibs that were judged in breeding shows (mean number of siblings: 188.5).

### Genomic data

In total, 3658 jumping horses, including jumping horses and stallions from previous studies [40–42], were genotyped with three single nucleotide polymorphism (SNP) density chips: the Illumina Equine SNP50 BeadChip that includes 54,602 SNPs (29% of the horses), the Illumina Equine SNP70 BeadChip that includes 65,157 SNPs (2% of the horses), and the Affymetrix Axiom Equine genotyping array that includes 670,806 SNPs (69% of the horses). Among these horses, 993 horses had morphometric data. During quality control, several SNPs were removed based on a minimum allele frequency test (MAF) < 5%, a Hardy–Weinberg test P-value < 10^–6^, a call rate < 90%, no valid position on an autosome, and P-value for the test of a difference in MAF between the three chips < 10^–5^. The EquCab 3.0 reference sequence was used to specify SNP positions on the reference map; EquCab 2.0 used as alternative reference when this information was not available (N = 2127 SNPs). After quality control, 375,687 SNPs were retained, which represented 73.2, 73.2, and 55.7% of the SNPs on, respectively, the Illumina Equine SNP50 chip, the Equine SNP70 BeadChip, and the Affymetrix Axiom Equine chip. Imputation was then performed using the Fimpute 3.0 software [43], and by adding pedigree information for four generations (18,682 horses). The genomic data were used only for genome-wide association analysis (GWAS).

### Genealogical data

Pedigree data were provided by the Institut Français du Cheval et de l’Equitation (IFCE), on behalf of the breeding organizations. Depending on the analysis, the number of horses in the pedigrees over six generations varied: 17,994, 28,270, 90,507, 331,062 horses for analyses that, respectively, involved only the morphometric data, the morphometric and the genomic data, the morphometric data and judges’ scores, and joint analysis with competition data.

### Morphometry analysis

**Fig. 3:**
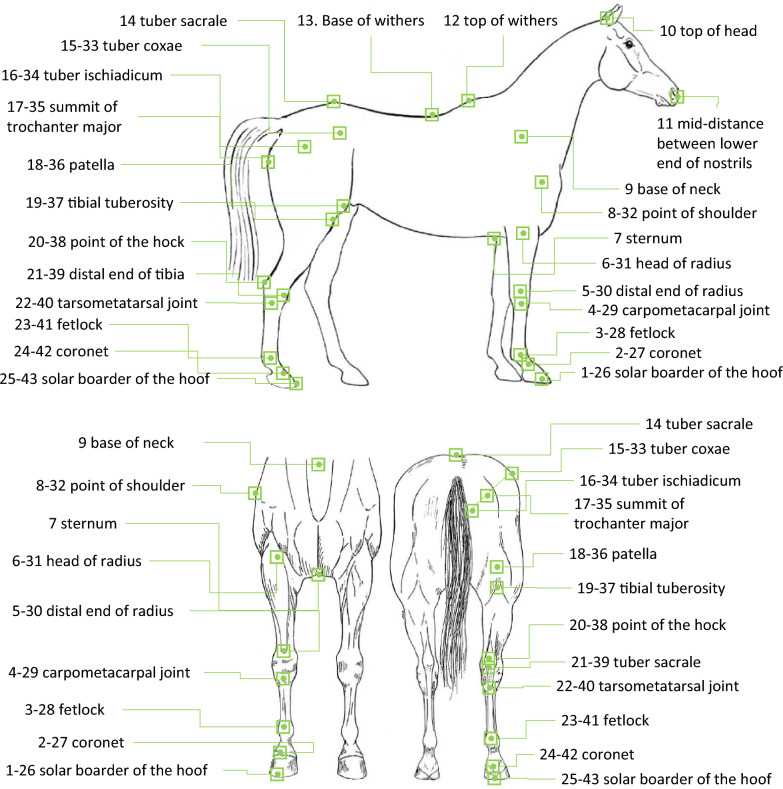
Anatomical landmarks used to define the morphology of the horse (right-left side)

Coordinates of the forehand and of the hindquarters reference images were combined to constitute the set of coordinates for a horse. These coordinates do not correspond to a real position of the horse because they were taken at different moments of the walking cycle, according to the position of the fore and hind cannon bone. Nevertheless, they allow a shape that is specific to the horse to be synthesized. Among the three redundant points between the two images, we chose to take two on the forelimb image: the top of the withers and the tuber sacral; and one on the hindlimb image: the tuber coxae. To obtain a three-dimensional shape, the landmarks collected from the right side were mirrored on the left side. For the neck and head landmarks, we retained the base of the neck, but the position of the top of the head and nostrils was standardized with a fixed angle between them and with the base of the neck. Because of differences in the walking phase when the image was selected, the actual position of the neck was not relevant for the morphological description. Each horse was therefore finally characterized by the three coordinates for 43 points (Fig. 3).

A Procrustes analysis was performed in order to center and scale the 3D landmark coordinates [34] using the Geomorph package [44] in the R environment, v. 4.0.2 [45]. The Procrustes analysis gave a centroid size and Procrustes coordinates. For the genetic analysis, only unconstrained variables were retained: Procrustes coordinates of points before mirroring and those that were not used as a reference or fixed by construction (for example, the X coordinate for point 4 was the same as that of point 3 because the verticality of the canon bone was used to define the reference image). In total, 63 coordinates were retained.

The Procrustes coordinates were analyzed using the following multitrait mixed model:

1 $${\mathbf{y}} = {\mathbf{Xb}} + {\mathbf{Zu}} + {\mathbf{e}},$$

where $\mathbf{y}$ is the vector of the coordinates ranked by horse; $\mathbf{b}$ is the vector of fixed effects including, for each coordinate, gender (female, male, gelding), age class (4 years and 5–6 years for young horses and > 6 years for stallions), operator (six levels), place and date of recording (24 levels), version of the protocol to identify landmarks (to account for slight changes in protocols over time, with three levels); $\mathbf{u}$ is the vector of random genetic effects, with $\mathbf{u}\sim N\left({\varvec{0}},\mathbf{A}\otimes \mathbf{G}\right),$ where $\mathbf{A}$ is the pedigree-based relationship matrix (for 17,994 horses) and $\mathbf{G}$ is the genetic variance–covariance matrix between coordinates; and $\mathbf{e}\sim N({\varvec{0}},\mathbf{I}\otimes \mathbf{R})$ is the vector of residuals, with $\mathbf{R}$ the residual variance–covariance matrix. The Wombat software [46] was used to estimate variance components by restricted maximum likelihood (REML). Bivariate analyses were performed for all possible pairs of the 63 coordinates. Then, estimates of 2 × 2 (co-)variance matrices from these 1953 analyses were combined to provide an overall 63 × 63 positive semi-definite (co-)variance matrix with the following requirements: elements ‘similar’ to those from individual analyses, no substantially change in estimates of variance ratios, and result in estimates of phenotypic covariances components that are little distorted. The method used is a likelihood-based approach which implicitly treats estimates from individual analyses as if they were matrices of corrected sums of squares and cross-products due to some pseudo observations [47]. The pool option of the software was used, with an equivalent number of half sib families of 580 with four offspring each.

Variance component estimates from the genetic analysis were illustrated by shapes, i.e., the eigenvectors $\mathbf{S}$ obtained from the eigen decomposition of the matrix $\mathbf{G}$: $\mathbf{G}=\mathbf{S}\Delta {\mathbf{S}}^{\mathbf{^{\prime}}}$. Then, summarized shapes for each horse were computed as linear combinations of Procrustes coordinates using the eigenvectors $\mathbf{S}$ as coefficients, i.e. as ${\mathbf{y}}_{\mathrm{i}}^{^\circ }=\mathbf{S}\mathbf{^{\prime}}{\mathbf{y}}_{\mathbf{i}}$ for horse i, where ${\mathbf{y}}_{\mathbf{i}}$ includes the 63 coordinates for horse $\mathrm{i}.$ Only the ten summarized shapes corresponding to the first ten principal components were used in further analyses. Heritabilities and genetic correlations between these shapes and centroid size were estimated using a bivariate analysis for centroid size with each shape $j=1\ldots 10$*,* ${y}_{ij}^{^\circ }$, using the model of Eq. (1). From these analyses, estimated breeding values of stallions ${\widehat{\mathbf{u}}}_{\mathrm{i}}^{^\circ }$ were obtained for the ten summarized shapes and visualized by plotting the coordinates obtained by back-transformation: ${\widehat{\mathbf{u}}}_{\mathbf{i}}=\mathbf{S}{\widehat{\mathbf{u}}}_{\mathrm{i}}^{^\circ }$.

### GWAS for morphometric shapes

A mixed linear animal model similar to Eq. 1 was used to analyze the summarized shapes ${\mathbf{y}}^{^\circ }$, using the BLUPF90 software [48]. The relationship matrix between genetic values was constructed using both pedigree and genomic data in a single-step genomic best linear unbiased prediction (GBLUP) method [49–52], as well as the rules outlined by [53] to construct the relationship matrix. GWAS was performed using back solutions of SNP effects and associated p-values, calculated as described in [54]. Genomic control [55] was applied to the p-values to prevent incorrect distribution of the test statistics. The effective number of independent tests in our data was calculated as the inverse of the mean of the linkage disequilibrium (r^2^) between all available pairs of SNPs by chromosome [56]. Linkage disequilibrium between chromosomes was assumed to be negligible. The genome-wide significance was then set at 1% and divided by the number of independent tests.

### Joint analysis of morphometry and performance in competition and judges scores

Bivariate models for each of the ten initial summarized shapes ${\mathbf{y}}^{^\circ }$ were used to estimate genetic correlations of morphometry with competition results and judges scores, using the Wombat software [46]. The model used for each ${\mathbf{y}}^{^\circ }$ was that of Eq. (1) with a supplementary conceptual permanent environmental effect to separate, in the residual, the part correlated with every performance in competition or breeding shows from the uncorrelated part. The model used for competition data and judges scores was:

2 $${\mathbf{y}}_{c} = {\mathbf{X}}_{c} {\mathbf{b}}_{c} + {\mathbf{Z}}_{c} {\mathbf{u}}_{c} + {\mathbf{W}}_{c} {\mathbf{p}}_{c} + {\mathbf{e}}_{c} ,$$

where ${\mathbf{y}}_{c}$ is the vector of annual competition performances or judges scores; ${\mathbf{b}}_{c}$ is the vector of fixed effects, the combined effect (134 levels) of year (from 2001 to 2019), age class (from 4 to 10 years old in steps of 1 year, 11–12 years old, and 13 years old and older), and gender (male: stallions and geldings together; female) for competition performance, and class of age (five levels according to age in days: ≤ 2.0 years, 2–2.5 years, 2.5–3 years, 3 years, 3.5 years, and > 3.5 years), year (from 2005 to 2019), local/regional/national type, category (six levels according to age in year and gender), gender (male/female), and show (1270 levels, same day and place) for judges scores; ${\mathbf{u}}_{c}$ is the vector of the breeding values, and ${\mathbf{p}}_{c}$ is the vector of permanent environmental effects common to the different years of competition performance or different breeding shows of judges scores of the same horse; ${\mathbf{X}}_{c}$, ${\mathbf{Z}}_{c},$ and ${\mathbf{W}}_{c}$ are the incidence matrices. The variance matrices were $V\left({\mathbf{u}}_{c}\right)=\mathbf{A}\otimes {\mathbf{G}}_{\mathbf{c}}$, $V({\mathbf{p}}_{c})=\mathbf{I}\otimes {\mathbf{P}}_{c}$ and $V\left({\mathbf{e}}_{c}\right)=\mathbf{I}\otimes {\mathbf{R}}_{c},$ where $\otimes$ is the direct product, $\mathbf{A}$ is the relationship matrix based on pedigree (331,062 horses for performance and 90,507 for judges’ scores), ${\mathbf{G}}_{c}$ the 2 × 2 genetic variance–covariance matrix for the two traits, ${\mathbf{P}}_{c}$ the 2 × 2 variance–covariance matrix for the permanent environmental effects of the two traits, $\mathbf{I}$ the identity matrix, and ${\mathbf{R}}_{c}$ the 2 × 2 diagonal residual variance–covariance matrix.

## Results

### Definition of shapes

**Fig. 4:**
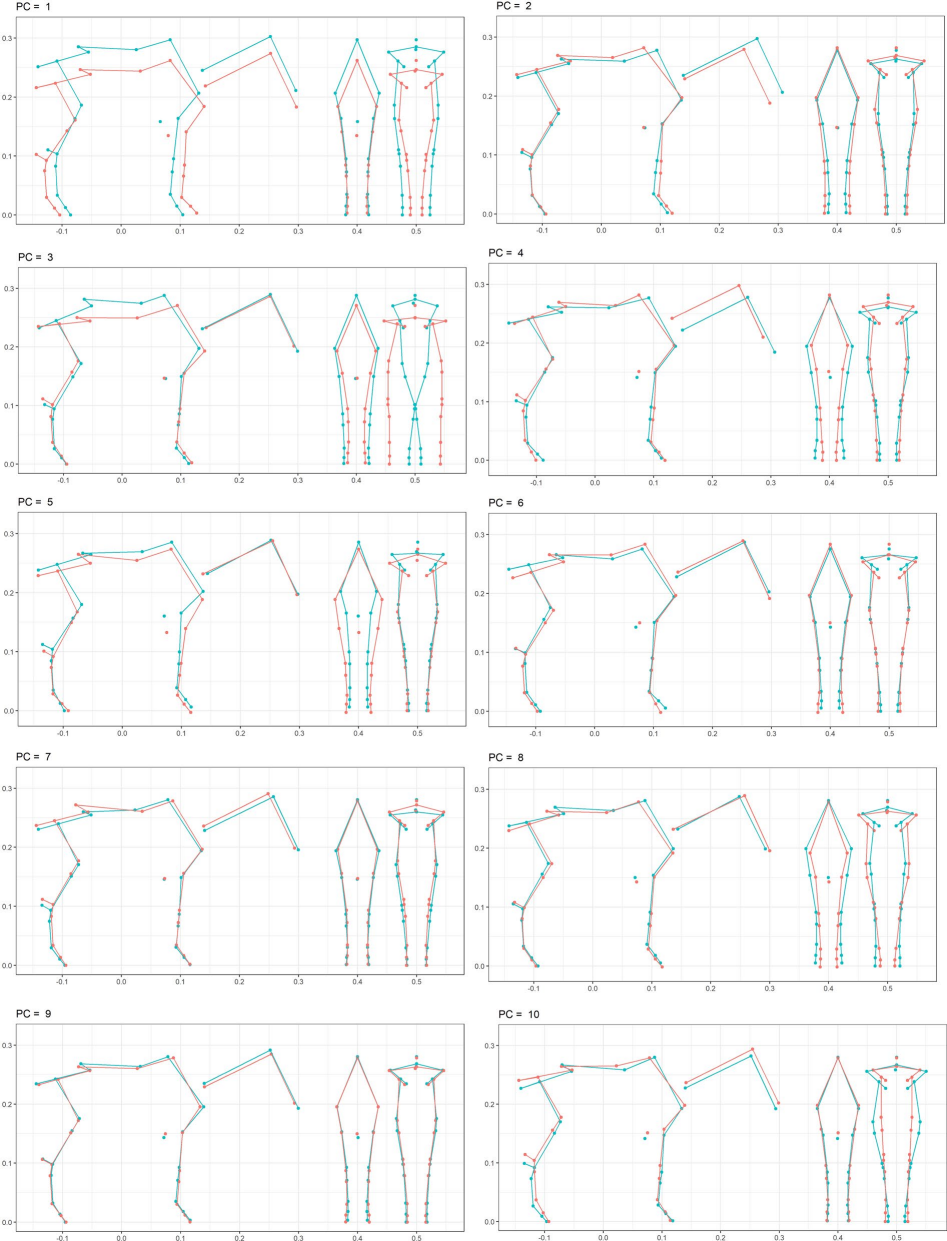
First ten shapes obtained by eigenvalue decomposition of the estimated genetic covariance matrix of Procrustes coordinates. Side view, front view forelimb, front view hindquarter; in blue, maximum positive deviation; in red, minimum

Elementary statistics on raw coordinates, judges’ scores, and competition performances are in Additional file 1 Table S1. Genetic analysis of the Procrustes coordinates estimated a genetic variance–covariance matrix. The proportion of genetic variance explained by the first component of the eigenvalue decomposition reached 31.9%. Eight components were needed to reach 80% and 13 to reach 90%. The percentage explained by the first ten components was 86%. The eigenvalue decomposition of this matrix resulted in eigenvectors, which are illustrated by the first ten shapes in Fig. 4. In this figure, the extreme shapes were defined by the observed maximum and minimum positions.

### Heritability of shapes

The ten shapes were estimated to be moderately to highly heritable (Table 1), especially shapes 1, 2, 5, 6, and 7 (estimates from 0.32 to 0.42). The estimated heritability of centroid size was 0.24 (se 0.07). Evidence of allometry was evident from estimates of genetic correlations between shapes and centroid size that were significantly different from 0 for shapes 1, 2, 7, and 10. A greater centroid size was associated with rectangular horses (small and long), long neck, hock behind, short forearm, stickle hock, inclined croup, inclined gaskin, and pronounced withers.

**Table 1:** Heritability estimates for the ten summarized shapes and their genetic correlation with centroid size (standard error)

Summarized shape	Heritability of shape	Heritability of centroid size	Genetic correlation	Phenotypic correlation
1	0.34 (0.07)	0.25 (0.07)	− 0.37 (0.15)	− 0.50 (0.02)
2	0.42 (0.07)	0.22 (0.06)	0.42 (0.16)	− 0.06 (0.02)
3	0.14 (0.05)	0.24 (0.07)	0.11 (0.23)	− 0.10 (0.02)
4	0.26 (0.06)	0.25 (0.07)	0.10 (0.19)	0.03 (0.02)
5	0.34 (0.07)	0.25 (0.07)	− 0.06 (0.18)	0.06 (0.02)
6	0.39 (0.07)	0.25 (0.07)	0.11 (0.17)	0.03 (0.02)
7	0.32 (0.06)	0.21 (0.06)	− 0.31 (0.18)	− 0.05 (0.02)
8	0.18 (0.06)	0.24 (0.07)	0.23 (0.21)	0.09 (0.02)
9	0.26 (0.07)	0.24 (0.07)	− 0.19 (0.19)	− 0.13 (0.02)
10	0.16 (0.05)	0.23 (0.06)	0.59 (0.20)	0.04 (0.02)

Summarized shapes were defined by the linear combination of 63 Procrustes coordinates using coefficients of the first ten eigenvectors of the genetic variance–covariance matrix of the coordinates. Procrustes coordinates and centroid size were obtained by morphometric analysis of major anatomical references from 3D views of the horse. Genetic variance–covariance matrix was estimated by pooling bivariate estimates from a sample of 2089 horses

### Genetic correlations with competition performance

The estimate of heritability of jumping performance was 0.30 (se 0.005) and its repeatability between years was equal to 0.51. Only one genetic correlation between summarized shapes and performance was estimated to be marginally significantly different from 0, i.e. + 0.12 for shape 2 (Table 2). Higher performance was genetically associated with a shape that combines a long neck, a high set of the neck (distant from the shoulder joint), straight and short shoulders, an oblique upper arm, a long back, a narrow croup, standing under for forelegs (side view), and with narrow forelimbs (front view). The estimate of the genetic correlation between jumping performance and centroid size was 0.33 (se 0.08).

**Table 2:** Heritability estimates for the ten summarized shapes and centroid size and of their phenotypic and genetic correlations with annual competition performance

Summarized shape	Heritability of shape^a^	Genetic correlation^a^	Phenotypic correlation^a^
1	0.35 (0.07)	0.01 (0.07)	0.01 (0.02)
2	0.43 (0.07)	0.12 (0.07)	0.02 (0.02)
3	0.14 (0.05)	− 0.08 (0.10)	0.00 (0.02)
4	0.26 (0.06)	− 0.08 (0.08)	0.01 (0.02)
5	0.34 (0.07)	0.01 (0.07)	− 0.04 (0.02)
6	0.40 (0.07)	0.05 (0.07)	0.00 (0.02)
7	0.34 (0.06)	− 0.04 (0.07)	0.00 (0.02)
8	0.18 (0.06)	0.08 (0.10)	0.04 (0.02)
9	0.28 (0.07)	0.10 (0.08)	− 0.02 (0.02)
10	0.17 (0.06)	0.04 (0.10)	− 0.04 (0.02)
Centroid size	0.25 (0.07)	0.33 (0.08)	0.06 (0.02)

For competition performance: h^2^ = 0.31; repeatability = 0.51, N = 150,817

Summarized shapes were defined by linear combination of 63 Procrustes coordinates using coefficients of the first ten eigenvectors of the genetic variance–covariance matrix of the coordinates. Procrustes coordinates and centroid size were obtained by morphometric analysis of major anatomical references from 3D views of the horse. Genetic variance–covariance matrix was estimated by pooling bivariate estimates from a sample of 2089 horses

^a^Standard error in brackets

### Genetic correlations with judges’ scores

The heritability estimates for the judges' scores (see Additional file 2 Table S2) were low for stance of forelegs (0.12, se 0.01) and stance of hindlegs (0.18, se 0.01), high for neck (0.38, se 0.02), impression (0.36, se 0.01) and chic (0.41, se 0.02), and moderate for the other scores (from 0.21 to 0.29). Estimates of genetic correlations between scores were all positive and high: from 0.51 to 0.92 (se from 0.003 to 0.040), but slightly lower between scores for stances, on the one hand, and body conformation, on the other hand. Estimates of genetic correlations scores with “impression” were very high for almost all scores (from 0.71 to 0.92).

**Fig. 5:**
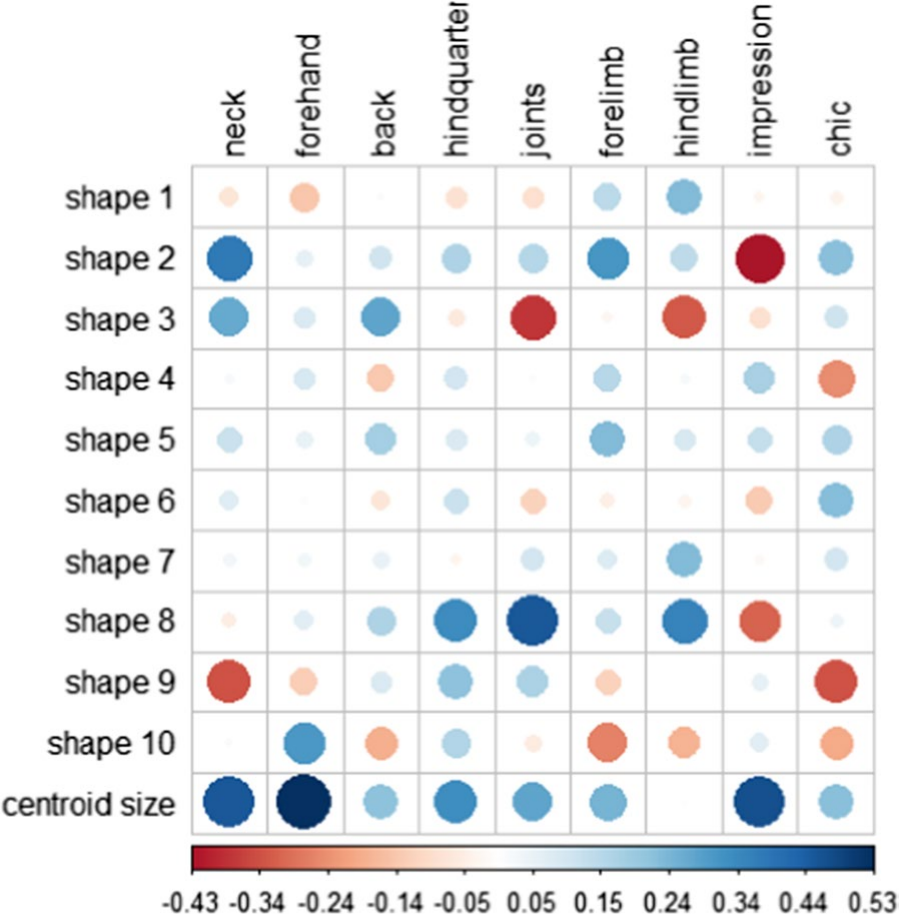
Estimates of genetic correlations between the ten summarized shapes, centroid sizes, and judges scores. Standard errors between 0.08 and 0.21

Figure 5 illustrates estimates of genetic correlations between judges’ scores and summarized shapes. Centroid size was estimated to be positively genetically correlated with each judges’ score, especially with forehand (0.53, se 0.11) and impression (0.46, se 0.10). Some shapes were more neutral for judges’ scores than others, i.e. shapes 1, 4, 5, 6, 7 and 10. For those shapes, only one or two of the nine genetic correlation estimates with judges’ scores were significantly different from 0 and did not exceed 0.31 (se 0.14). Table 3 lists the main physical characteristics derived from the observation of shapes that were favorably correlated genetically with the scores awarded by the judges. There was no shape for which genetic correlation estimates had the same sign with all judges’ scores; judges were apparently looking for a combination of morphologic characteristics that do not naturally genetically combine in horses.

**Table 3:** Description of morphological traits that were favorably scored by judges according to estimates of genetic correlations between judges’ scores^a^ and summarized shapes

Score	Morphological trait favourably scored	Shapes involved
Neck	Long neck, short head	2, 3, 9
Body forehand	Straight shoulders, large elbow angle and long thoracic depth	10
Withers-back-loins	Short back, short withers, dipped line of the back	3
Body hindquarters	Long, narrow and flat croup and long gaskin length	8
Joints	Large joints, short stifle and long pasterns	3, 8
Stance of forelegs	Standing under for forelegs (side view) and narrow forelimbs (front view), long forelimbs and long and straight pasterns	2, 10
Stance of hindlegs	Wide base of hindleg, long hind pasterns and large joints	3, 8
Impression	Wither located behind the shoulder and uphill line of the back	2, 8
Chic	Small head	9

Summarized shapes were defined by the linear combination of 63 Procrustes coordinates using coefficients of the first ten eigenvectors of the genetic variance–covariance matrix of the coordinates. Procrustes coordinates and centroid size were obtained by morphometric analysis of major anatomical references from 3D views of the horse. Genetic variance–covariance matrix was estimated by pooling bivariate estimates from a sample of 2089 horses

^a^Judges’ scores recorded on 28,412 horses

### Genome-wide association study of summarized shapes

Based on the mean linkage disequilibrium across the genome of 0.00034822, the estimated number of independent tests of 1/0.00034822 = 2872. The p-value threshold was then set to 1%/2872 = 3.5 × 10–6 or − log(p-value) = 5.46. Genomic control was applied to ensure a regression coefficient close to 1 between ranked observed log(p-value) on expected log(p-value) in the QQ plots (see Additional file 3 Figure S1). For centroid size, we found large numbers of significant SNPs on chromosome 3 between 106,477,263 and 108,336,766 bp, with the largest log(p-value) of 38.2 for rs394972354 (at 107,643,810 bp), and one other region on chromosome 1 between 55,808,413 and 60,105,801 bp, with the largest − log(p-value) of 8.6 for rs1145108512 (see Additional file 3 Figure S1). For all shapes, several SNPs were found to be significant (Table 4): one for shapes 1, 3, 4, 6, and 10, two for shape 2, and three for shape 9, with none overlapping between shapes.

**Table 4:** Significant SNPs for summarized shapes

Shape number	Chromosome	Physical position (bp)	db SNP ID	Allele (Ref./Alt.)	MAF	Log (p-value)^a^
1	19	50,554,884	rs1137772803	A/G	0.50	5.83
2	19	35,110,857	rs69216956	A/G	0.33	6.63
2	19	35,111,080	rs69216958	G/T	0.33	6.64
3	1	144,952,487	rs1151909758	A/G	0.07	5.67
4	3	107,272,714	rs395965848	G/A	0.29	6.64
6	22	36,493,364	rs395833721	G/A	0.48	5.49
9	1	15,716,473	rs1149598923	A/G	0.08	5.86
9	1	66,305,171	rs1148287010	C/T	0.17	5.58
9	28	40,962,032	rs69354804	C/T	0.38	5.48
10	1	101,966,349	rs68605628	A/G	0.06	5.71

*MAF* minimum allele frequency

^a^Corrected by genomic control

### Breeding values of stallions

**Fig. 6:**
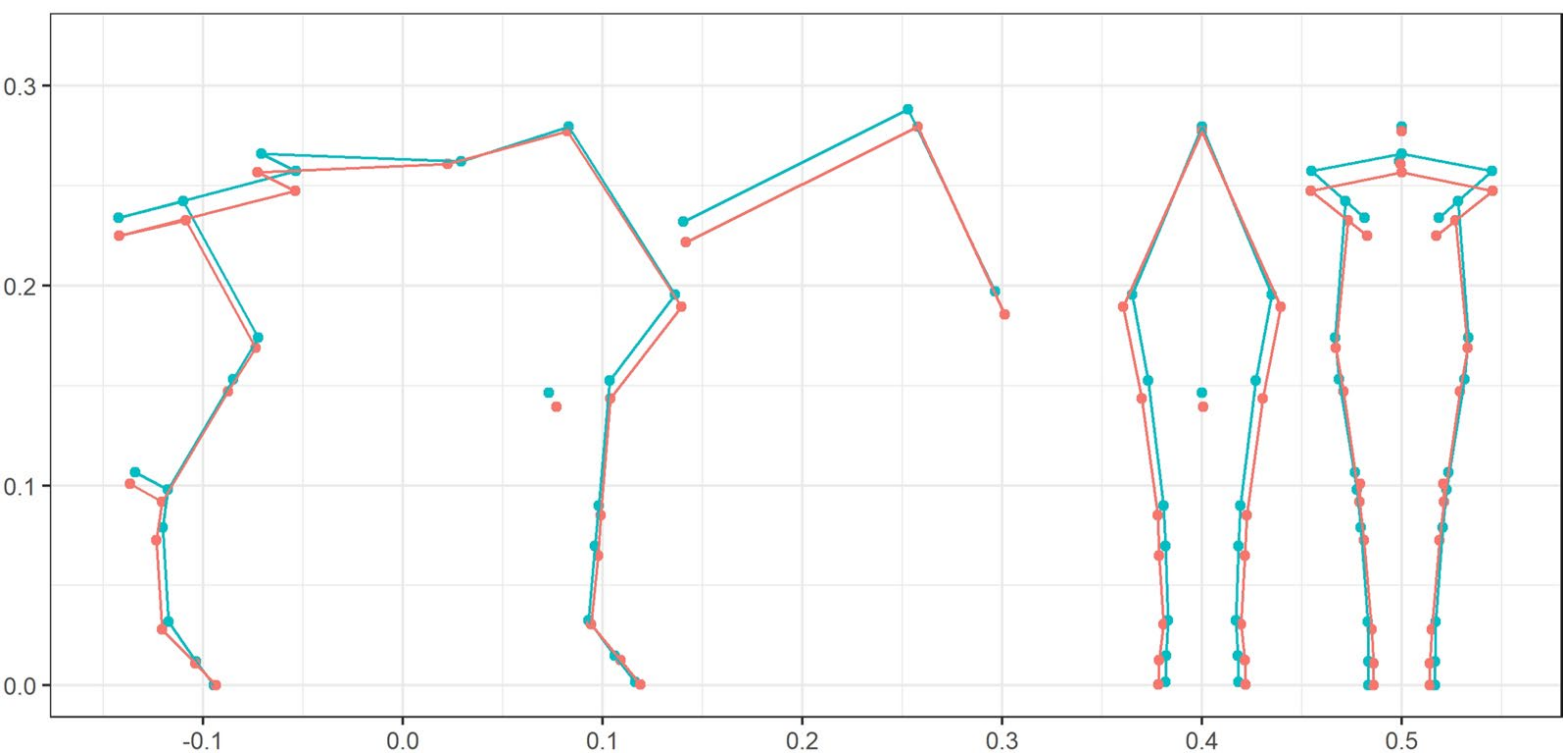
Estimated breeding value of the stallion APACHE D’ADRIERS (SF). Estimated breeding values in red compared to the population mean in blue

Estimated breeding values (EBV) for stallions and mares were calculated for the ten summarized shapes. Then, the ten EBV were converted to the 63 coordinates, which were used to create a single image as the expected shape of progeny, which is an attractive way to present EBV for breeders. An example for the famous stallion APACHE D’ADRIERS is in Fig. 6.

## Discussion

### Special features of genetic analysis of geometric morphometrics

Morphology of an organism must be studied in the form of synthetic criteria, which are the combinations of the coordinates of the elementary points placed anatomically on the organism. However, the possible number of anatomical landmarks used to describe morphology is necessarily large. In our case, we recorded the position in space (three coordinates) of 25 points, i.e., 75 variables. Since the position of some was fixed beforehand, 63 variables remained. Estimating the variance–covariance matrix of 63 variables is a challenge since it involves 2016 genetic (co-)variances and as many residuals (co-)variances. In order to estimate these variance components while reducing the size of the system, Meyer [46] developed an algorithm and a software program, WOMBAT, that performs an eigenvalue and eigenvector decomposition of the genetic and residual variance–covariance matrix simultaneously with likelihood maximization of the variables subject to the fitted linear mixed model. However, because the software is limited to 34 variables, this option was therefore not possible. As stated by Meyer [47], “estimation of large variance–covariance genetic matrices is computationally highly demanding and can be afflicted by convergence problems”. Estimating the covariances in pairs solved the problem, especially since Meyer developed a method to bend the different estimates into a definite positive matrix based on a penalized likelihood approach [57, 58] and showed by simulations [47] that this strategy was closer to a complete multivariate analysis than alternatives that considered overlapping subsets of traits. On the basis of the estimated genetic variance–covariance matrix, we were able to derive the eigenvectors of the main shapes. Genetic analysis of shapes has already been performed in papers on evolutionary studies with fewer variables [35, 59, 60], and we showed that this was also possible for a more complex morphology.

### Geometric morphometric analysis of horses

Cervantes et al. [61] were the first to introduce geometric morphometrics and generalized Procrustes analysis (GPA) to characterize the morphology of horses. Using 2D images from 171 Spanish Arab horses, they used nine landmarks on major joints of the body. Later, Druml et al. [14, 62] defined the shape of 44 Lipizzan horses based on 2D images using 246 landmarks that covered the shape of the horse, including head, neck, and legs. The same procedure was applied to 243 Franches-Montagnes stallions [12]. Those studies were limited to phenotypic analysis. The first genetic study was performed by Gmel et al. [36] on 608 horses. They estimated heritabilities for shapes defined by principal component analysis (PCA) following phenotypic GPA. Estimates of heritability of the five first shapes ranged from 0.13 to 0.37. We defined shapes using PCA on the estimated genetic variance–covariance matrix rather than on the phenotypic variance–covariance matrix. David et al. [63] showed that defining summarized phenotypes based on eigenvectors of the genetic (co-)variance matrix for genetic analysis of longitudinal data gave breeding values of similar quality than summarized breeding values and was easier to compute in a routine way. In this article, the quality of EBV was assessed for accuracy and bias using partial and whole data by selecting certain generations. For our data, it is difficult to routinely estimate the genetic values of 63 coordinates and provide summarized breeding values. In the same way than David et al. [63], it is much simpler to compute once the genetic (co-)variance matrix between the 63 coordinates and then to use routinely the top ten summarized shapes to provide EBV for sires and dams. We have not compared EBV obtained from the analysis of summarized shapes with summarized breeding values obtained from the analysis of the 63 coordinates but we expect the same efficiency for the quality of prediction than in David et al. [63]. We expect that summarizing shapes defined by eigenvectors will be more efficient to predict unbiased and accurate morphology of progeny when based on estimated genetic versus phenotypic (co-)variance matrices, as was done by [36], because the genetic instead of the phenotypic correlations are taken into account. In both cases, estimates of heritability of shapes were found to be low to moderate.

### Traditional conformation evaluation and geometric morphometrics

Many studies have reported genetic parameters on conformation traits for horses. Traits were either based on subjective judgments, linear profiling, or objective measurements and horses were from different breeds and types (draught horses, local breeds, sport and leisure horses). This variety can easily explain the variability in genetic parameters reported in the meta-analysis of [64]. Geometric morphometrics highlight the dependency between the characteristics of the different parts of the body. As an illustration of this, estimates of genetic correlations between linear profiling traits that describe each morphological zone have varied widely, ranging from negative to positive, for several jumping horse breeds [20, 22, 23, 27, 65], as well as for draught horses [4–7] and other sports or leisure horses [9, 10, 15–17]. It is not possible to select for one criterion without changing the overall conformation of the horse. Defining shapes is a way to reduce the number of features needed to describe a morphology. The objective of reducing the number of features can also be pursued in the analysis of linear profiling or objective measurements. For this purpose, factor analysis of conformation traits has been carried out by [28], 66. This is more interesting for linear profiling than for judges’ scores because estimates of genetic correlations for judges’ scores are very often high and positive, implying that the judgment of each part of the body may be subjectively influenced by the overall feeling that the horses’ conformation is good or bad [24, 25, 42, 65, 67, 68], except quite often for leg conformation, which is more specific.

Our estimates of genetic correlations between judges’ scores and shapes indicate that the conformation desired by judges does not correspond to conformation determined by genetic correlations between anatomical landmarks. This is in agreement with the findings of [62], who failed to find a regression between shapes and judges’ scores. There are several possible explanations for these findings. The first is that the judgement-based ideal conformation combines traits from different parts of the body that are not genetically positively correlated. In this case, the selection objective of judges can still be reached, but it is more difficult. The second is that the judges’ scale gives the maximum score for an intermediate value between two anatomical extremes. If the shape is based on the anatomical difference between these two extremes, the genetic correlation between judges score and shape will be low, even if it is the same trait. This was reported by [26] between judges scores and linear profiling traits. For example, they estimated a high genetic correlation between judges’ scores and the linear profiling trait ‘neck’ scored arched vs. straight, but a low genetic correlation for loins scored long vs. short. In the first case, the desired trait is an extreme, i.e. an arched neck, whereas in the second case the desired trait is an intermediate, i.e. an intermediate size loin. The third explanation may be that the judges idea of a ‘good’ horse’ may vary over time [24] and we have captured the different trends over time in the correlation.

### Correlation between conformation and jumping performance

Previous estimates of genetic correlations of conformation linear profiling traits or judges’ scores with jumping competition results are low. In the Netherlands, Rovere et al. [69] estimated a genetic correlation of 0.23 of jumping results with judges’ conformation scores (an update of the estimate of 0.29 in [21]) and Koenen et al. [20] estimated generally non-significant genetic correlation of jumping results with linear profiling traits, with a maximum of 0.28 for muscularity of haunches. In Sweden, Jönsson et al. [70] estimated genetic correlations ranging from 0.13 to 0.30 between objective measurements and judges’ scores of conformation traits during riding horse quality tests and lifetime performances, and Viklund et al. [71] estimated genetic correlations ranging from 0.19 to 0.22 of the same traits with competition results measured by points and placings. In Germany, Stock et al. [72] estimated correlations between EBV of linear profiling traits of conformation with jumping competition traits that ranged from -0.14 to 0.21. In Denmark, Seierø et al. [73] estimated genetic correlations between judges’ scores and two jumping competition criteria based on success (accumulated lifetime points) and longevity (number of active years in competition) that generally were not significantly different from zero, with a maximum of 0.23 for hindlimb scores. Our results agree with these literature results. Biologically, conformation is related to functionality and the absence of a strong genetic correlation between morphology and competitive performance may be unexpected. First, the methodology that only takes correlation into account may not have been able to find a complex non-linear relationship between morphology and jumping performance, regardless of whether morphology was measured by shapes, as in our study, or by linear profiling, as in the literature. Second, the morphology in the populations studied, which have been selected for jumping results for several generations, may have reached the minimum conformation requirement needed and thus the differences in competition could be due to many other traits, such as physiology, mental, ability to learn, and resistance to stress. Therefore, morphology may not be a major determinant of jumping success for breeds that are already selected for sports.

### Genomic analysis

We did not find any significant SNPs in the regions that were reported in [74] for conformation traits for Franche Montagne and Lipizzan traits. With the exception of height at withers, we also found no SNPs in the regions of known major genes, even those known to be related to conformation. For example, we found no SNPs in the region of the Warmblood Fragile Foal Syndrome (WFFS), known to be linked to conformation traits [75]. Significant SNPs associated with centroid size on chromosome 3 were in the region of the *LCORL* gene, which is known to be linked to height at withers [76–80]. These SNPs were not significantly linked to the first shape, which distinguishes “rectangular” from “square” horses. Since a “square” horse is taller than he is long, this region could be assumed to be associated with this shape. However, this was not the case: thus this region is likely associated with overall development of the horse, i.e., the centroid size, and not specifically with height at withers when this measurement is considered proportionally to the others. Using standardized shapes of the horse relative to volume has enabled us to separate the biological mechanism that is responsible for overall size from the mechanism that is responsible for shape, as already reported by Makvandi-Nejad et al. [77], who performed a GWAS on the first principal component of the phenotypic analysis of 33 measurements on various breeds. This principal component involved all measurements and was defined as body size information. Both the square/rectangle shape and the centroid size were moderately heritable in our data.

## Conclusions

Conformation of the horse has been studied for a very long time. Genetic analysis of generalized Procrustes coordinates avoids the challenging problem of describing and judging conformation. It also allowed us to eliminate the volume effect, which impacts any other subjective or objective conformation traits. In particular, we found that the known quantitative trait locus linked to the *LCORL* gene is not associated with shape of the horse (greater than long) but with its overall development. We propose to routinely use ten summarized shapes defined as linear combinations of Procrustes coordinates using the ten first eigenvectors of genetic (co-)variance matrix of coordinates. We found no significant genetic correlation of shapes with jumping performance but conformation remains a selection objective in itself, which will be facilitated by estimated breeding values presented as a single attractive image of the shape of future progeny. An image is easy to understand but the synthetic and complex aspect of the summarized shape may be difficult to handle for breeders, thus its analytical translation into the language traditionally used in breeding may be necessary.

### Supplementary Information

**Additional file 1: Table S1.** Elementary statistics of raw coordinates, judges’ scores and competition performances. This table contains three sheets for raw coordinates, judges’ scores and competition performance, respectively, and provides the number of records, mean, standard deviation, minimum and maximum of all variables used in the analysis.

**Additional file 2: Table S2.** Heritability and genetic correlations between judges’ scores.

**Additional file 3: Figure S1.** QQ plot and Manhattan plot of GWAS for summarized shapes and centroid size.

## Data Availability

These data (genotypes and phenotypes) are part of a reference population used for genomic selection and have commercial value. Therefore, restrictions apply to their availability, and they are not publicly available. The authors can be contacted for a reasonable request.
