# Supplementary material for: Genetic analysis of geometric morphometric 3D visuals of French jumping horses
Source: Genet Sel Evol. 2023 Sep 18;55:63. doi: 10.1186/s12711-023-00837-8 (PMC10506242; doi:10.1186/s12711-023-00837-8)
Supplement: Supplementary file 3 — Additional file 3: Figure S1. QQ plot and Manhattan plot of GWAS for summarized shapes and centroid size. [file 12711_2023_837_MOESM3_ESM.pptx]

## Slide 1
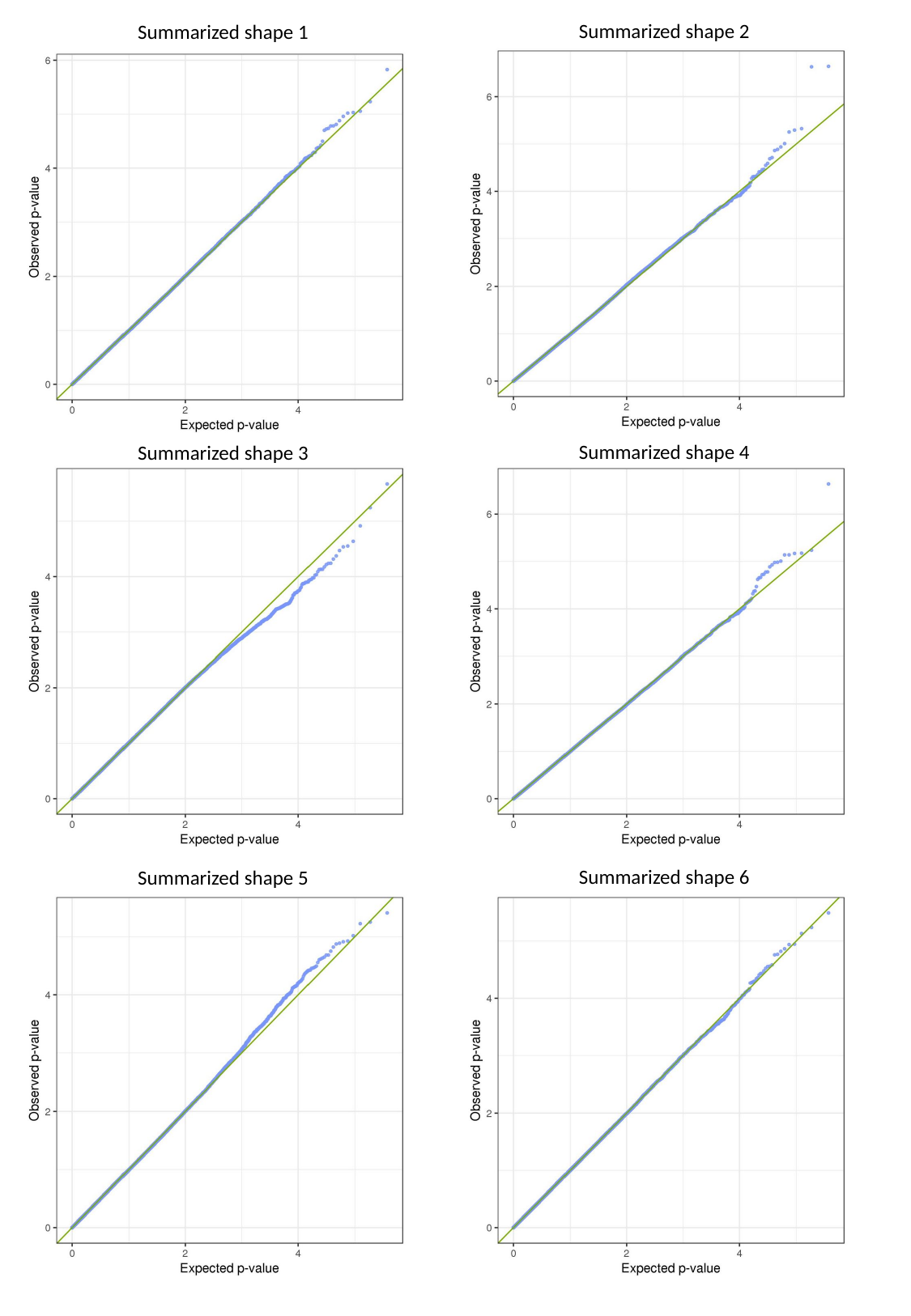

Summarized shape 2
Summarized shape 1
Summarized shape 4
Summarized shape 3
Summarized shape 6
Summarized shape 5

## Slide 2
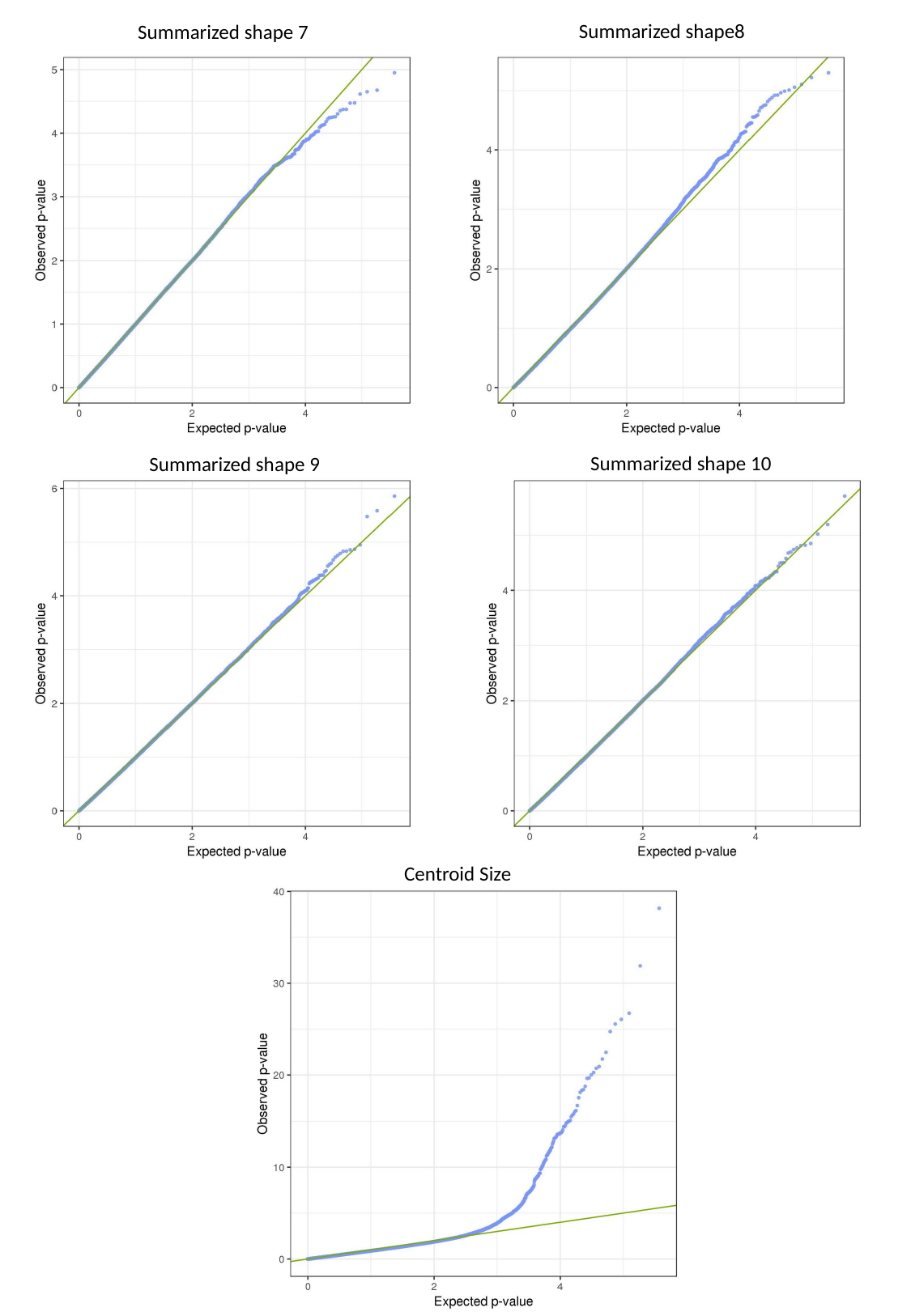

Summarized shape8
Summarized shape 7
Summarized shape 10
Summarized shape 9
Centroid Size

## Slide 3
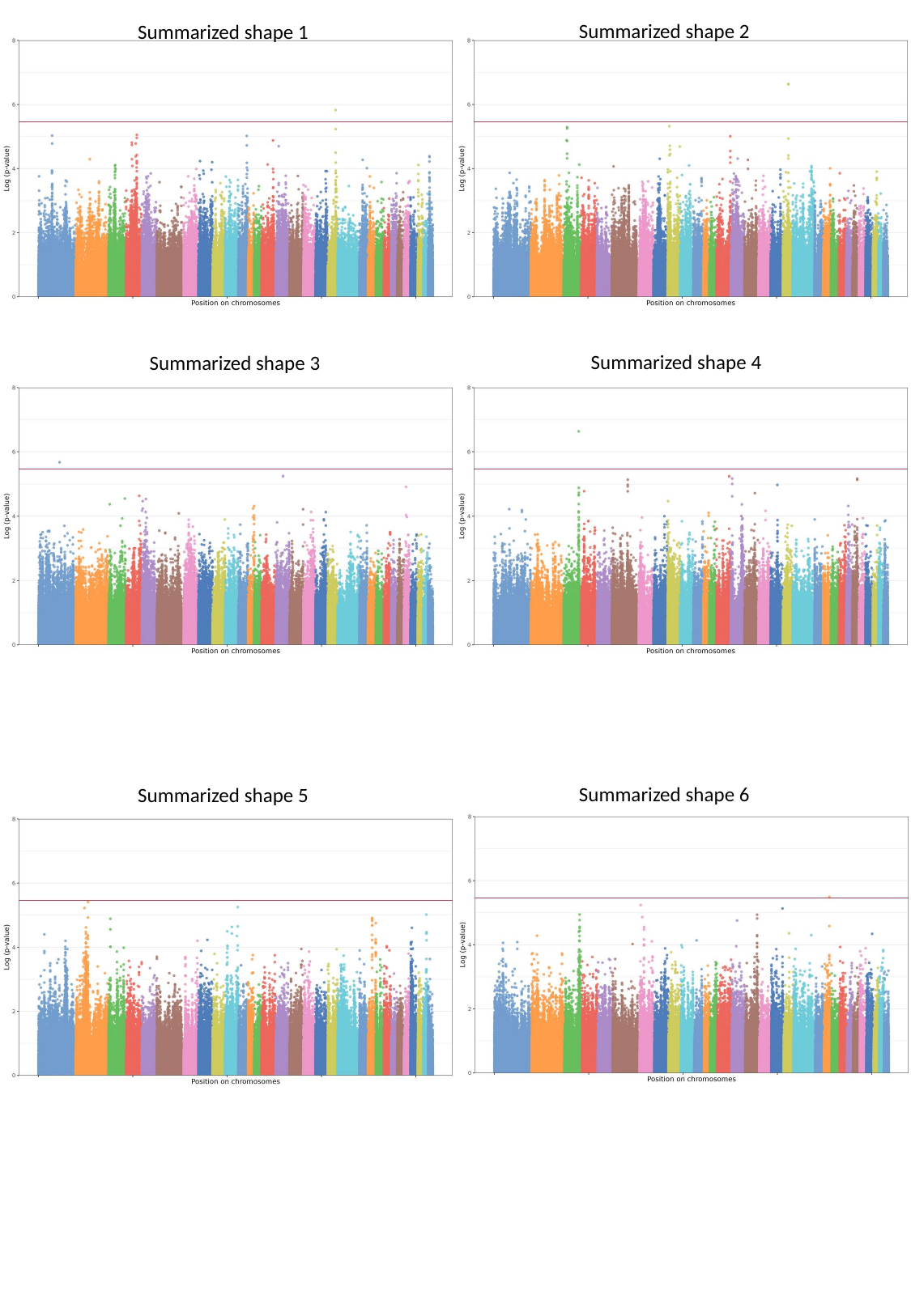

Summarized shape 2
Summarized shape 1
Summarized shape 4
Summarized shape 3
Summarized shape 6
Summarized shape 5

## Slide 4
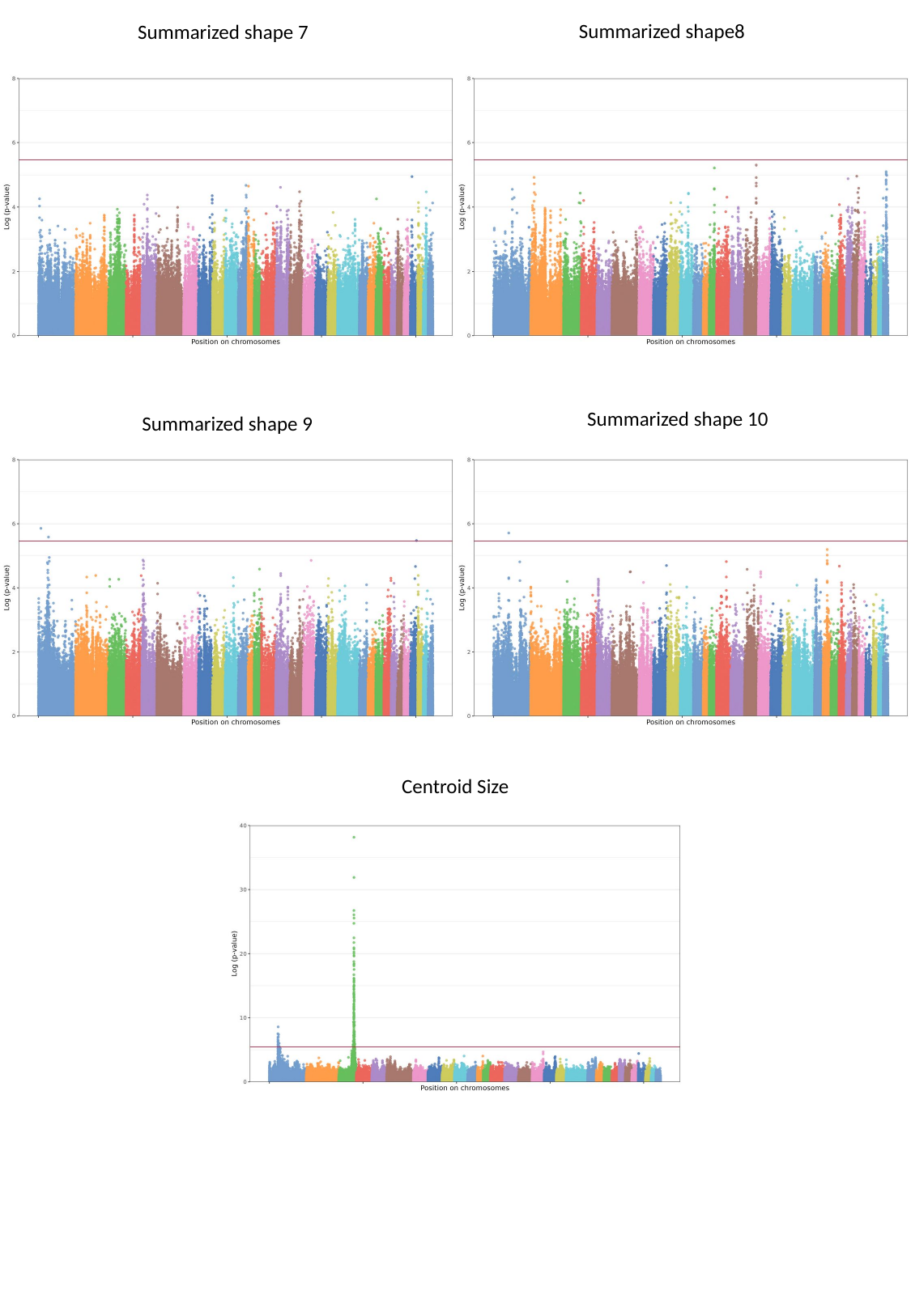

Summarized shape8
Summarized shape 7
Summarized shape 10
Summarized shape 9
Centroid Size
